# Analysis of trends in adolescent suicides and accidental deaths in England and Wales, 1972–2011

**DOI:** 10.1192/bjp.bp.114.162347

**Published:** 2016-10

**Authors:** James Redmore, Ruth Kipping, Adam Trickey, Margaret T. May, David Gunnell

**Affiliations:** **James Redmore**, BSc, MPH, **Ruth Kipping**, MA(Cantab), MSc, MA(Lond), PhD, FFPH, **Adam Trickey**, BSc, MSc, **Margaret T. May**, MA(Cantab), MSc, PhD, **David Gunnell**, MB ChB, PhD, FFPH, MRCGP, MFPHM, DSc, FMedSci, School of Social and Community Medicine, University of Bristol, Bristol, UK

## Abstract

**Background**

Previous analyses of adolescent suicides in England and Wales have focused on short time periods.

**Aims**

To investigate trends in suicide and accidental deaths in adolescents between 1972 and 2011.

**Method**

Time trend analysis of rates of suicides and deaths from accidental poisoning and hanging in 10- to 19-year-olds by age, gender and deprivation. Rate ratios were estimated for 1982–1991, 1992–2001 and 2002–2011 with 1972–1981 as comparator.

**Results**

Suicide rates have remained stable in 10- to 14-year-olds, with strong evidence for a reduction in accidental deaths. In males aged 15–19, suicide rates peaked in 2001 before declining. Suicide by hanging is the most common method of suicide. Rates were higher in males and in 15- to 19-year-olds living in more deprived areas.

**Conclusions**

Suicide rates in adolescents are at their lowest since the early 1970s with no clear evidence that changes in coroners' practices underlie this trend.

Suicide is one of the leading causes of preventable death in adolescents worldwide, and is ranked second in terms of the number of potential life years lost in those aged 20–54 years within the UK.^1^ Suicide rates are not routinely reported for 10- to 14-year-olds in the UK and rates for 15- to 19-year-olds are often combined with those for 20- to 24-year-olds, masking trends in the younger ages. Furthermore, combining rates fails to reflect the differing physiological, social and psychological developmental stages of adolescents and young adults.^2^

With a few exceptions, previous analyses of suicides in those aged 10–19 years have focused on discrete time periods and have used date of death registration as opposed to date of actual death.^3–6^ Further, no study has investigated whether suicide rates in adolescents differ depending on levels of deprivation in England.

Recent concerns about possible influences of suicide rates in young people include regulatory activity to restrict antidepressant prescribing,^7^ the 2008 economic recession^8^ and the potential of internet use to encourage suicidal behaviour.^9^ Our study aimed to report numbers, rates and trends of suicide (suicide and undetermined deaths) and accidental poisoning and hanging deaths in adolescents aged 10–19 years by gender across four decades: 1972–1981, 1982–1991, 1992–2001 and 2002–2011. In addition, we report analysis of suicide by socioeconomic deprivation for 15–19 year olds in England during 2002–2011.

## Method

We defined adolescents as aged 10–19 years.^10^ The setting of the study was England and Wales, 1972–2011.

### Suicide, undetermined and accidental poisoning and hanging deaths data

We obtained suicide mortality data for males and females in England and Wales from 1972 to 2011, which were registered by 31 December 2012, from the Office for National Statistics (ONS; personal communication). Data were based on the actual year of death, as opposed to date of death registration. This distinction is potentially important as date of death registration may post-date the death by 1 year or more because of delays in completing inquests. Classification of deaths for this study used ICD-8 (deaths registered from 1972 to 1978),^11^ ICD-9 (deaths registered from 1979 to 2000)^12^ and ICD-10 (deaths registered from 2001 to 2012).^13^ Deaths with the following final underlying cause were included: (a) intentional self-harm (ICD-8 and ICD-9: E950–E959, ICD-10: X60–X84 and Y87.0), (b) injury/poisoning of undetermined intent (ICD-8 and ICD-9: E980–E989, ICD-10: Y10–Y34 and Y87.2, excluding ICD-9 code E988.8 for the years 1979–2000, and ICD-10 code Y33.9 for the years 2001–2006 as these latter codes were used when the coroners' verdicts were pending). It is conventional practice for government suicide statistics in the UK to combine suicide and undetermined intent deaths, as most deaths of undetermined intent are thought to be suicides when reviewed by clinicians.^14^ For simplicity we refer to combined deaths categorised as suicide or of undetermined intent as ‘suicides’ throughout this paper. In addition, we investigated trends in accidental poisoning by solids, liquids and gases (referred to as accidental poisoning hereafter; ICD-8: E850–E877, ICD-9: E850–E869, ICD-10: X40–X49) and deaths as a result of accidental hanging, strangulation and suffocation (referred to as accidental hanging hereafter; ICD-8 and ICD-9: E913, ICD-10: W75–W77, W81, W83, W84) to account for potential misclassifications of suicides because of coroner's growing use of narrative verdicts.^15^

Data were available by age at death in years (10–19 years), gender, method of suicide/undetermined/accidental death (via specific ICD codes), and Index of Multiple Deprivation (IMD) decile (2001–2011, for England only). See online Table DS1 for a full list of ICD codes for method-specific suicides and accidental poisoning and hanging deaths, corresponding to the three ICD versions used in our analyses.

### Population data

We used mid-year population estimates for England and Wales provided by the ONS (personal communication), which have been revised to take account of the most recent 2011 census, to calculate age-specific suicide rates (10–14 years, and 15–19 years) by gender. IMD decile-specific mid-year population estimates for England only were used to calculate suicide rates by IMD decile. For ease of interpreting trends, we collapsed the IMD deciles into quintiles.

### Index of Multiple Deprivation

The IMD for England is derived from economic, social and housing indicators combined into a unified score for each small area.^16^ The IMD deciles for Wales are not comparable with those of England. Further, the number of adolescent suicides from 2001 to 2011 in Wales was too small to conduct country-specific analysis using IMD scores, as was the data availability for 10- to 14-year-olds in England. Therefore, analyses of suicide in relation to socioeconomic deprivation were restricted to 15- to 19-year-olds in England, which comprised 91% of all suicides. Analysis was carried out using Stata version 13.1, unless otherwise stated.

### Statistical methods

We used Poisson models to estimate rate ratios for suicide/undetermined deaths and accidental (poisoning and hanging) deaths for 1982–1991, 1992–2001 and 2002–2011 with 1972–1981 as comparator. To test for linear trend in the outcomes, the year of death was entered in the model as a continuous variable. We checked for overdispersion using a negative binomial model and compared goodness-of-fit using the log-likelihood. All analyses were stratified by gender and age at death (10–14 and 15–19 years).

## Results

Between 1972 and 2011 there were 7001 suicide deaths among those aged 10–19 years, of whom 580 (8%) were aged 10–14 years. Suicide deaths were more frequent in males than females, with a male to female gender ratio of 1.9:1.0 in those aged 10–14 years, and 2.7:1.0 in those aged 15–19 years. Between 1972 and 2011, there were 3006 accidental poisoning and hanging deaths among those aged 10–19 years. Those aged 10–14 years accounted for 817 (27%) of all accidental deaths in 10–19 year olds.

Table 1 displays the number and rates of method-specific suicide and accidental poisoning and hanging deaths, by gender, in those aged 10–14 years and 15–19 years, respectively, for each of the four decades we analysed. Hanging and solid and liquid poisoning were the two most frequent methods of suicide in all age and gender groups throughout 1972–2011. Suicide by gas poisoning was rare in 10- to 14-year-olds of both genders across our study period. In males and females aged 15–19 years, rates of suicide by gas poisoning and by jumping increased from 1972 to 1991 before decreasing between 1992 and 2011, with the decrease in suicides by gas poisoning being particularly large. More detailed gender and age-specific trends in suicide and accidental poisoning and hanging deaths are presented below.

**Table 1:** Numbers and rates of method-specific suicide deaths and accidental deaths in males and females aged 10–14 years and 15–19 years in England and Wales, 1972 to 2011

	10–14 years^a^				15–19 years^b^			
	1972–1981 *n* (rate)	1982–1991 *n* (rate)	1992–2001 *n* (rate)	2002–2011 *n* (rate)	1972–1981 *n* (rate)	1982–1991 *n* (rate)	1992–2001 *n* (rate)	2002–2011 *n* (rate)
*Suicide deaths^c^*								
Solid or liquid poisoning								
Male	13 (0.06)	0(0)	7 (0.04)	3 (0.02)	276 (1.44)	208 (1.08)	198 (1.26)	73 (0.42)
Female	33 (0.17)	16 (0.10)	23 (0.14)	12 (0.07)	348 (1.91)	239 (1.31)	175 (1.15)	86 (0.51)
Gas poisoning								
Male	<5^d^	<5^d^	0(0)	0(0)	79 (0.40)	218 (1.15)	106 (0.68)	13 (0.07)
Female	<5^d^	<5^d^	0 (0)	0 (0)	5 (0.03)	27 (0.15)	14 (0.09)	<5^d^
Hanging								
Male	63 (0.31)	57 (0.35)	76 (0.46)	67 (0.39)	272 (1.41)	508 (2.68)	607 (3.84)	627 (3.54)
Female	6 (0.03)	8 (0.05)	18 (0.11)	41 (0.25)	40 (0.22)	67 (0.37)	105 (0.69)	204 (1.20)
Drowning								
Male	13 (0.06)	5 (0.03)	0 (0)	<5^d^	62 (0.33)	58 (0.30)	46 (0.29)	31 (0.18)
Female	<5^d^	0(0)	<5^d^	0 (0)	24 (0.13)	9 (0.05)	7 (0.05)	6 (0.04)
Firearms and explosives								
Male	8 (0.04)	<5^d^	<5^d^	<5^d^	92 (0.47)	118 (0.61)	45 (0.29)	25 (0.14)
Female	<5^d^	<5^d^	0 (0)	0 (0)	11 (0.06)	5 (0.03)	<5^d^	0(0)
Cutting or piercing								
Male	0 (0)	<5^d^	0 (0)	0 (0)	7 (0.04)	11 (0.06)	8 (0.05)	5 (0.03)
Female	0 (0)	0 (0)	0 (0)	0 (0)	<5^d^	<5^d^	<5^d^	0(0)
Jumping or falling								
Male	5 (0.02)	<5^d^	<5^d^	0 (0)	86 (0.45)	137 (0.71)	60 (0.38)	46 (0.26)
Female	<5^d^	<5^d^	<5^d^	0 (0)	37 (0.20)	45 (0.24)	20 (0.13)	16 (0.10)
Other or unspecified methods								
Male	19 (0.09)	12 (0.07)	8 (0.05)	5 (0.03)	147 (0.75)	180 (0.94)	223 (1.42)	163 (0.93)
Female	5 (0.03)	6 (0.04)	6 (0.04)	<5^d^	41 (0.22)	40 (0.21)	57 (0.37)	45 (0.27)

*Accidental deaths*								
Accidental poisoning								
Male	47 (0.23)	36 (0.20)	18 (0.11)	17 (0.10)	252 (1.31)	265 (1.36)	374 (2.38)	208 (1.17)
Female	37 (0.19)	35 (0.22)	18 (0.11)	15 (0.09)	152 (0.83)	122 (0.67)	131 (0.86)	120 (0.71)
Accidental hanging								
Male	190 (0.93)	124 (0.73)	121 (0.73)	105 (0.81)	172 (0.89)	143 (0.73)	96 (0.61)	102 (0.58)
Female	11 (0.06)	6 (0.04)	12 (0.07)	25 (0.15)	6 (0.04)	8 (0.04)	11 (0.07)	27 (0.16)

a. Rates are per 100000 population aged 10–14 years.

b. Rates are per 100000 population aged 15–19 years.

c. Includes undetermined verdicts.

d. Cells contain between 1 and 4 deaths and are censored.

### Trends in suicide and accidental deaths in England and Wales

#### Males aged 10–14 years

Between 1972 and 2011 there were 385 suicide deaths (range: 3–18 annually; mean rate of 0.55 deaths per 100 000 population), 540 accidental hanging deaths (range: 4–27 annually; mean rate of 0.75 per 100 000 population) and 118 accidental poisoning deaths (range: 0–10 annually; mean rate of 0.16 per 100 000 population) among males aged 10–14 years.

**Fig. 1:**
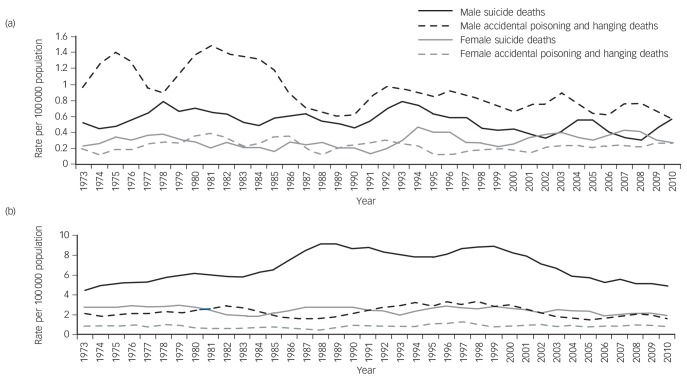
Three-year moving averages of rates (per 100 000) of suicide (suicide and undetermined) deaths and accidental (poisoning and hanging) deaths for males and females aged: (a) 10–14 years and (b) 15–19 years in England and Wales, 1972–2011.

The overall mean rate of suicide in males aged 10–14 years was 0.60 per 100 000 between 1972 and 1981 and 0.45 per 100 000 between 2002 and 2011 (Fig. 1(a)), rate ratio 0.75 (95% CI 0.56–1.00). There was no statistical evidence of a trend in the incidence of suicide across the years of the study period (*P* trend 0.34, Table 2). Hanging was the most common method in males aged 10–14 years, accounting for 68% of all suicide deaths. Online Fig. DS1(a) highlights mean rates per decade of suicide by solid and liquid poisoning and hanging. Rates of suicide by hanging increased slightly, from 0.31 per 100 000 between 1972 and 1981 to 0.39 per 100 000 between 2002 and 2011.

**Table 2:** Rate ratios (95% CI) comparing 1972–1981 *v.* 1982–1991, 1992–2001, 2002–2011 and log-likelihoods and *P*-values of a continuous year term^a^

	Male				Female			
	Suicide/undetermined		Accidental		Suicide/undetermined		Accidental	
	Aged 10–14	Aged 15–19	Aged 10–14	Aged 15–19	Aged 10–14	Aged 15–19	Aged 10–14	Aged 15–19
Decade								
1972–1981: rate ratio, reference	1	1	1	1	1	1	1	1
1982–1991: rate ratio (95% CI)	0.90 (0.69–1.18)	1.39 (1.28–1.50)	0.83 (0.68–1.01)	0.95 (0.83–1.09)	0.81 (0.53–1.24)	0.85 (0.75–0.97)	1.04 (0.69–1.58)	0.84 (0.67–1.06)
1992–2001: rate ratio (95% CI)	0.95 (0.73–1.24)	1.54 (1.42–1.67)	0.72 (0.58–0.88)	1.35 (1.18–1.54)	1.10 (0.75–1.62)	0.90 (0.79–1.03)	0.78 (0.50–1.23)	1.10 (0.88–1.38)
2002–2011: rate ratio (95% CI)	0.75 (0.56–1.00)	1.04 (0.95–1.13)	0.61 (0.49–0.76)	0.79 (0.68–0.91)	1.26 (0.87–1.83)	0.76 (0.67–0.87)	0.98 (0.64–1.49)	1.02 (0.82–1.28)

Continuous year term, Poisson								
Log-likelihood	−110.7	−271.4	−125.1	−171.8	−88.9	−143.1	−87.9	−128.4
*P*	0.268	<0.001	<0.001	0.221	0.079	0.004	0.526	0.275

Continuous year term, negative binomial								
Log-likelihood	−109.6	−194.4	−122.4	−155.3	−88.9	−141.7	−87.7	−124.7
*P*	0.341	0.118	<0.001	0.513	0.079	0.014	0.554	0.422

a. Analyses by death type, gender and age groups. Poisson models were compared with negative binomial models for the continuous year term.

Accidental hanging accounted for 82% of all the accidental deaths studied in this age group. There has been a reduction in rates of accidental hanging and poisoning deaths, rate ratio 0.61 (95% CI 0.49–0.76) for 1972–1981 *v.* 2002–2011, which provides evidence that the slight decline in suicide rates is not as a result of changing coroners' verdicts in favour of accidental deaths. The death rate because of accidental hanging fell, from 0.93 per 100 000 between 1972 and 1981 to 0.81 per 100 000 between 2002 and 2011 (Table 1). The death rate as a result of accidental poisoning fell, from 0.23 per 100 000 between 1972 and 1981 to 0.10 deaths per 100 000 between 2002 and 2011.

#### Females aged 10–14 years

Between 1972 and 2011 there were 195 suicide deaths (range: 1–10 annually; mean rate of 0.29 per 100 000 population), 54 accidental hanging deaths (range: 0–4 annually; mean rate of 0.08 per 100 000 population) and 105 accidental poisoning deaths (range: 0–8 annually; mean rate of 0.15 per 100 000 population) in females aged 10–14 years.

Suicide rates remained steady across our study period, from a mean rate of 0.28 per 100 000 between 1972 and 1981 to 0.35 per 100 000 between 2002 and 2011, rate ratio 1.26 (95% CI 0.87–1.83). In the first three decades (1972–2001), solid and liquid poisoning was the most common method of suicide in females aged 10–14 years (mean 0.14 deaths per 100 000). This has been replaced with suicide by hanging in the most recent decade (2002–2011, mean 0.25 deaths per 100 000; online Fig. DS1(a) and Table 1).

Accidental death rates overall have fluctuated slightly in each of the four decades across our study period, but no clear trend was evident (mean rate of 0.25 per 100 000 between 1972 and 1981 compared with a mean rate of 0.24 per 100 000 between 2002 and 2011), rate ratio 0.98 (95% CI 0.64–1.49). There has been a reduction in accidental poisoning deaths, from 0.19 per 100 000 between 1972 and 1981 to 0.09 per 100 000 between 2002 and 2011. This has been accompanied by an increase in the rate of deaths by accidental hanging across the study period, from 0.06 per 100 000 between 1972 and 1981 to 0.15 per 100 000 between 2002 and 2011 (Table 1).

#### Males aged 15–19 years

Between 1972 and 2011 there were 4735 suicide deaths (range: 67–179 annually; mean rate of 6.70 per 100 000 population), 513 accidental hanging deaths (range: 3–30 annually, mean rate of 0.70 per 100 000 population) and 1099 accidental poisoning deaths (range: 13–54 annually; mean rate of 1.56 per 100 000 population) in males aged 15–19 years.

Suicide rates rose from 3.10 deaths per 100 000 in 1972 to a peak of 9.10 deaths per 100 000 in 1989, before gradually reducing to a current low of 4.80 deaths per 100 000 in 2011 (Fig. 1(b)). Compared with 1972–1981, the rate ratio was 1.54 (95% CI 1.42–1.67) and 1.04 (95% CI 0.95–1.13) in 1992–2001 and 2002–2011, respectively. Hanging was the most common method of suicide in males aged 15–19 years, accounting for 43% of suicide deaths in this age group (Table 1 and Fig. 1(b)).

Rates of suicide by hanging have more than doubled in the past 40 years, from a mean rate of 1.41 deaths per 100 000 between 1972 and 1981 to a mean rate of 3.54 deaths per 100 000 between 2002 and 2011. A large reduction in suicides by solid or liquid poisoning is evident, particularly throughout the most recent decade, with rates falling from 1.26 deaths per 100 000 between 1992 and 2001 to 0.42 deaths per 100 000 between 2002 and 2011. Rates of suicide by gas poisoning also fell from 0.40 deaths per 100 000 between 1972 and 1981 to 0.07 deaths per 100 000 between 2002 and 2011. Reductions in the rate of suicide by drowning, firearms and explosives, and jumping/falling from high places are evident across our study period (Table 1).

Accidental death rates fluctuated between 1.50 and 3.32 deaths per 100 000 between 1972 and 2011 (Fig. 1(b)). Compared with 1972–1981, the rate ratio for accidental deaths was 1.35 (95% CI 1.18–1.54) and 0.79 (95% CI 0.68–0.91) in 1992–2001 and 2002–2011, respectively. Accidental poisoning was the most common method of accidental death in males aged 15–19 years, accounting for 68% of all accidental deaths. Rates of accidental poisoning increased from 1972 to 2001 (1.68 deaths per 100 000) before falling between 2002 and 2011 (1.17 per 100 000). Rates of accidental hanging deaths have generally fallen in males aged 15–19 years across the study period (Table 1).

#### Females aged 15–19 years

Between 1972 and 2011 there were 1686 suicide deaths (range: 24–65 annually; mean rate of 2.48 per 100 000 population), 52 accidental hanging deaths (range: 0–7 annually, mean rate of 0.08 per 100 000 population) and 525 accidental poisoning deaths (range: 4–26 annually; mean rate of 0.77 per 100 000 population) in females aged 15–19 years.

There was a reduction in the rate of suicide, from 2.82 per 100 000 between 1972 and 1981 to 2.16 per 100 000 between 2002 and 2011 (Fig. 1(b)), rate ratio 0.76 (95% CI 0.67–0.87). Solid and liquid poisoning was the most common method of suicide between 1972 and 2001 (mean 1.46 deaths per 100 000). This has been replaced with suicide by hanging, with a mean rate of 1.20 deaths per 100 000 between 2002 and 2011 (Table 1 and online Fig. DS1(b)). The rate of suicide by solid or liquid and gas poisoning has fallen throughout each decade, whereas the rate of suicide by hanging increased fivefold from 1972 to 2011.

Accidental death rates fluctuated between 0.21 and 1.49 deaths per 100 000 between 1972 and 2011 (Fig. 1(b)). Rates of accidental poisoning deaths remained relatively stable across the study period. Rates of death by accidental hanging increased slightly across the study period, particularly in the most recent decade (Table 1). However, overall, there has been little change in the rate of accidental deaths across the study period, rate ratio 1.02 (95% CI 0.82–1.28) comparing 2002–2011 with 1972–1981. Trends in suicide rates in all age groups were broadly similar when accidental death and suicide were combined (see online Fig. DS2).

### Suicide and deprivation in England

**Fig. 2:**
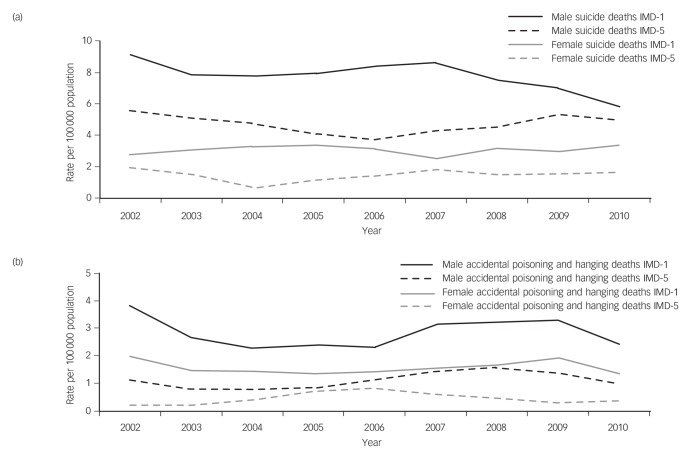
Three-year moving averages of rates (per 100 000) of: (a) suicide (suicide and undetermined) deaths and (b) accidental (poisoning and hanging) deaths by Index of Multiple Deprivation (IMD) Quintile (1, most deprived, 5, least deprived) for males and females aged 15–19 years in England, 2001 to 2011.

Figure 2 displays 3-year moving average rates of (a) suicide and (b) accidental poisoning and hanging deaths, centred on the middle year, from 2001 to 2011, by IMD quintile (lowest *v.* highest), in males and females aged 15–19 years. When comparing the mean rate of suicide from 2001 to 2011, those in the most deprived IMD quintile had a 79% higher rate of suicide (10.90 per 100 000) compared with those in the least deprived IMD quintile (6.10 per 100 000). There is some evidence of this gap narrowing in the latter part of the decade. This social gradient is higher (threefold increase) for accidental deaths in males and females aged 15–19 years (4.69 per 100 000 in the most deprived IMD quintile, 1.55 per 100 000 in the least deprived IMD quintile).

## Discussion

Overall, rates of suicide in both males and females aged 10–14 years remained relatively stable throughout the past 40 years. The rate of suicide in males aged 15–19 years steadily rose between 1972 and 2001, before declining in 2002–2011, although there is evidence that this decline has plateaued since 2006. In older adolescent females, there has been a moderate fall in the rate of suicide from 1972 to 2011. Our findings are broadly in line with the trends in child and adolescent suicide and accidental deaths reported by McClure from 1970 to 1998,^4^ and Windfuhr from 2001 to 2010.^6^ One particularly striking finding was the extent to which hanging has replaced self-poisoning as the most common method of suicide in both 10–14 and 15–19 year old females between 2002 and 2011. This replicates the pattern observed in adolescents in the USA^17^ and other countries.^18^

The fall in accidental hanging and poisoning deaths in recent years suggests there has been no major shift in the categorisation of suicides deaths as accidents in those aged 10–19 years following the growth in use of narrative verdicts in the 2000s. Therefore, the generally downward trends in suicide are likely to be genuine rather than an artefact of coding changes.

Our analysis of suicide rates by deprivation emphasises the social gradient of suicide among adolescents. Recent data from the Scottish Suicide Information Database showed a similar pattern in young adults.^19^ In Northern Ireland, a disproportionate rise in the rate of suicide in adolescents aged 12–17 years was also observed between the most deprived compared with the least deprived 20% of areas.^20^

We found an overall gender ratio for male to female suicide deaths of 1.9:1.0 in 10- to 14-year-olds and 2.7:1.0 in 15- to 19-year-olds. The gender paradox of self-harm and suicide has been widely reported in the literature, both within the UK and worldwide.^21^ Differences in the acceptability of various methods of suicide may account for divergent suicide death ratios between genders. Females have a greater preponderance to self-harm,^22^ but tend to use less lethal suicide methods compared with males^23^ although the rise in suicides by hanging in females signals a possible change in this pattern.

In our analysis, accidental deaths by hanging and poisoning accounted for 58% of all deaths in those aged 10–14 years, compared with 25% in the 15–19 year age group. Most strikingly the number of accidental hanging deaths in boys aged 10–14 exceeded suicides by 40.3%. In 10–14 year olds, the ratio of accidents to suicide is higher than in 15–19 year olds. This may indicate that possible suicides in 10–14 year olds are more likely to receive an accidental verdict and suicide statistics are substantially underestimated. The capability of younger adolescents to take their own lives has been widely debated in the literature.^24^ Coroners may feel pressured by family members and others to record a verdict other than suicide.^15^

The World Health Organization Mortality Database has been used to examine trends in rates of suicide in both 10- to 14-year-olds^25^ and 15- to 19-year-olds^26^ in recent years. Suicide rates in those aged 10–14 years remained relatively stable between 1990 and 2009 in most countries, with the exception of some Eastern European and South American countries, where rates have increased significantly. For 15- to 19-year-olds, trends in rates were higher in non-European countries and in males in the majority of countries worldwide.

### Factors influencing suicide rates over the past 40 years

The availability and associated lethality of a range of suicide methods changed throughout our study period. Rates of suicide by gas poisoning rose from the early 1970s until 1992, before rapidly declining thereafter. This can be ascribed to the mandatory fitting of catalytic convertors in newly manufactured petrol motor vehicles from 1993 onwards.^27^ More recently, reductions in the rate of suicide by solid and liquid poisoning may partially be explained by legislation to reduce the pack size of paracetamol and salicylates in 1998,^28^ the withdrawal of co-proxamol sales in 2005^29^ and the transition in the prescribing of high-toxicity tricyclic to lower-toxicity selective serotonin re-uptake inhibitor (SSRI) antidepressants.^30^ We found no clear evidence of a rise in suicides from 2003 onwards. This is reassuring given the Medicines and Healthcare Products Regulatory Agency activity to restrict prescribing of SSRIs to those aged below 18 years as a result of limited evidence of effectiveness and a concern that they may increase the risk of suicidal behaviour.^7^ There was a substantial reduction in suicide deaths by jumping from the mid-1990s onwards for both males and females aged 15–19 years. This may have been because of the implementation of structural interventions to reduce the means of individuals jumping from notable suicide hot spots.^31^

Despite relatively large reductions in suicides by gas poisoning, and solid and liquid poisoning, it is clear that suicide by hanging has partially substituted these and other methods of suicide over the past 40 years. This applies to all adolescents and is of particular concern because of the high case fatality rate of hanging as a method of suicide, and the relatively limited approaches available to professionals in preventing deaths by hanging.^32^ Our study period contained four periods of official recession: in the mid-1970s, early 1980s, early 1990s and most recently in the late 2000s.^33^ There is no clear evidence in our analysis that these influenced suicide rates in 10–19 year olds.

### Strengths and limitations

This is the first comprehensive long-term investigation of trends in suicide deaths in adolescents in England and Wales. We were also able to explore the impact of deprivation on suicide rates in those aged 15–19 years. Although high-quality, national-level data were obtained, there are a number of limitations in our analysis.

First, the quality of reporting and its subsequent impact upon coding accuracy is an issue. There are widely divergent views in the literature regarding the capacity of younger adolescents aged 10–14 to (a) understand the concept of suicide and (b) intentionally end life. Consequently, accurate suicide rates in those aged 10–14 years, and to a lesser degree those aged 15–19 years, may be masked by a classification of some self-inflicted deaths as accidental, as opposed to suicide.^14^ We addressed this by including accidental deaths by hanging and poisoning in our analyses. Second, our study period saw two changes in ICD classifications used; one in 1990 (ICD-8 to ICD-9) and another in 2001 (ICD-9 to ICD-10). No discontinuities were evident when visually inspecting suicide rates and trends around these two periods. Further, previous analyses have shown that changes between versions have not influenced overall suicide rates.^34^

Third, some ICD code-specific methods of accidental poisoning (such as E850.0, X42; drugs of misuse, including opiates) and accidental hanging (such as W77; threat to breathing due to cave-in, falling earth and other substances) that have been included in our analyses are more likely to be genuine accidents rather than misclassified suicides. For accidental poisoning deaths, drugs of misuse accounted for around one-third of deaths (mostly in those aged 15–19 years), and there was evidence of a rise in deaths as a result of opiate use in the late 1990s. However, removal of these deaths in a sensitivity analysis had little impact on the observed trends. For accidental hanging, deaths coded as W77 are rare (<2% of accidental hanging deaths overall) and have a negligible effect on overall trends. A small number of accidental hanging deaths may also be attributed to a phenomenon known as the ‘Choking Game’.^35^ Fourth, the number of suicide and accidental deaths in those aged 10–14 years was very small, with some years having no cases. This resulted in fluctuating rates, which were partially countered by using 3-year rolling averages centred on the middle year, and the calculation of weighted mean rates by decade. However, trends in this age group should be interpreted with caution.

### Implications

Our analysis has provided further detail concerning rates and trends of various suicide methods in recent years. Of particular note is the increase in rates of suicide by hanging in all age groups across the past 40 years, particularly in females. The National Suicide Prevention Strategy for England places an emphasis on restricting means to access suicide.^36^ Given the continual increase in suicide by hanging in both younger and older adolescents, and the fact that only a small proportion of deaths by this method are amenable to method restriction^32^ there needs to be an increased emphasis on improving mental health of young people in relevant settings, such as schools.^37^ Negating the perception young people have of hanging as a ‘quick and easy death’ may also help.^38^

A recent report highlights the significant gaps in local implementation of the National Suicide Prevention Strategy for England.^39^ It is essential that local areas have a suicide prevention action plan, a multiagency suicide prevention group and audit local suicides.^40^ In addition, the systematic method of reviewing all child deaths, as is undertaken in England and Wales, Australia, New Zealand and the USA, is an important method of identifying opportunities to prevent future child deaths.^41,42^

An analysis of adolescent suicides in England between 1997 and 2003 found only 14% of those dying by suicide had contact with mental health services in the year prior to death, compared with around 26% of the adult population.^6^ Although the majority of adolescents will have experienced mental health problems prior to suicide, in excess of 20% do not. Thus, there is a need for universal mental health promotion and suicide prevention strategies at multiple levels, including that of the individual, family, school, employer, media and community.
